# A method for mechanically blocking antennal joints in *Drosophila*

**DOI:** 10.1242/jeb.251495

**Published:** 2025-11-21

**Authors:** Olivia M. Nunn, Tobias C. McCabe, Kevin M. Mills, Weizhi Cao, Marie P. Suver

**Affiliations:** ^1^ Department of Biological Sciences, Vanderbilt University, Nashville, TN 37210, USA; ^2^ Vanderbilt Brain Institute, Vanderbilt University, Nashville, TN 37210, USA

**Keywords:** *Drosophila*, Antenna, Mechanosensory, Glue, Johnston's organ, Active sensing

## Abstract

Mechanosensation provides an animal with proprioceptive information about the body's motion and position, and exteroceptive information such as sounds and tactile signals. Insects use chordotonal organs to convert mechanical forces (e.g. airflow and substrate vibrations) into electrical signals that the brain can integrate with other sensations and regulate ongoing motor output. In *Drosophila melanogaster*, the largest chordotonal organ, composed of about 1000 primary mechanoreceptors, is housed in the antennae. These peripheral sensory neurons detect tonic and phasic changes in the position of the third antennal segment relative to the second, enabling detection of airflow and sound to guide behaviors such as locomotion and courtship. Here, we demonstrate how to precisely apply glue to antennal joints to mechanically block these stretch-mediated receptors. This technique can be used in studies aimed at understanding the contribution of antennal mechanosensation and/or the influence of active antennal movements for sensation and behavior.

## Teaser

**Summary:** Segmented antennae house sensory neurons and muscles crucial for sensing; these sensory appendages can be precisely immobilized using this detailed protocol, which effectively blocks joint movement and sensation in *Drosophila*.

## INTRODUCTION

Insects rely on three primary types of mechanoreceptors – chordotonal organs, campaniform sensilla and bristles – to detect and process mechanical stimuli. These receptors, distributed across the body, allow insects to perceive and integrate sensory cues such as load, posture, movement, sound, substrate vibrations, wind and touch. This mechanosensory input enables the detection of external tactile stimuli (exteroception) while simultaneously shaping the organism's perception of its own position and movement (proprioception). Although these receptors are located across the entire body, the antennae serve as a key mechanosensory appendage where all three types are found.

**Fig. 1.**
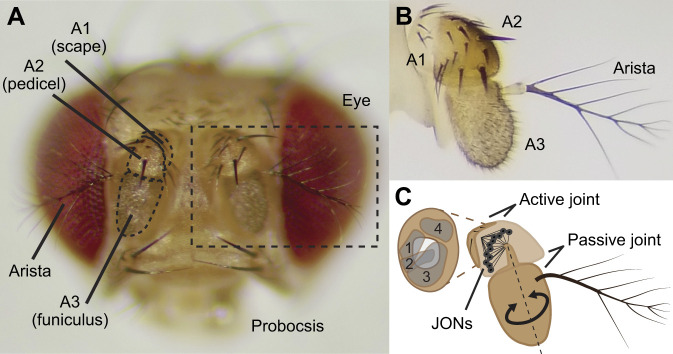
**Anatomy of the *Drosophila* antenna.** (A) Frontal view of the head. Two segmented antennae are located on the anterior side of the head. Labels indicate names of the first (A1, scape), second (A2, pedicel) and third (A3, funiculus) segments of the antennae. (B) Lateral image of the left antenna attached to the head capsule (boxed area in A) (adapted from Suver et al., 2023). A1 attaches to the head, A2 is directly attached to A1, and A3 inserts into A2 and forms the funicular joint; the arista is attached to A3. (C) Schematic diagram of internal antennal anatomy. A1 contains four antennal muscle groups (gray, labeled 1–4) and attaches to A2, forming the active antennal joint. A2 contains Johnston organ neurons (JONs) that attach to the funicular hook on A3 and are activated by movements of the passive joint.

*Drosophila melanogaster* antennae (Fig. 1) house all three primary types of mechanoreceptors and serve as a mobile sensory structure actuated by a set of muscles housed in the first segment (Miller, 1950; Suver et al., 2019). These muscles create an active joint between the scape (first antennal segment; A1) and the pedicel (second antennal segment; A2). In contrast, the joint between the pedicel and the funiculus (third antennal segment; A3) is not directly moved by any muscle. Instead, this joint and A3 are passively moved either by active motion at the A1–A2 joint or by small fluctuations in air particle velocity, with the arista amplifying its mechanical impact (Göpfert and Robert, 2001). These movements are transduced in A2 by about 1000 mechanoreceptors (Johnston, 1855; Kamikouchi et al., 2006), called Johnston organ neurons (JONs) – which together compromise the largest mechanosensory organ in the animal, the Johnston's organ. JONs convert mechanical forces acting on the antennae into electrical signals, enabling the brain to encode stimulus features such as frequency, amplitude and phase (Dieudonné et al., 2014; Göpfert and Robert, 2001; Heinzel and Gewecke, 1987; Patella and Wilson, 2018). Many studies in several insect species have established that JONs contribute to behaviors such as sound localization (Batchelor and Wilson, 2019; Gibson and Russell, 2006), courtship (Baker et al., 2022; Burnet et al., 1971; Charlwood and Jones, 1979; Ejima and Griffith, 2008; Manning, 1967; Tootoonian et al., 2012), steering (Camhi and Johnson, 1999; Natesan et al., 2019), head stabilization (Chatterjee et al., 2022), grooming (Hampel et al., 2015, 2020; Zhang et al., 2020), odor gradient tracking (Duistermars et al., 2009), wing motor reflexes (Mamiya et al., 2011; Mamiya and Dickinson, 2015), flight speed regulation (Fuller et al., 2014), wind encoding (Chang et al., 2016; Okubo et al., 2020; Suver et al., 2019, 2023), gravity sensing and locomotion (Budick et al., 2007; Kamikouchi et al., 2009; Robie et al., 2010; Sun et al., 2009; Yorozu et al., 2009). Crucially, many of the major conclusions from these past studies were supported by experiments that used glue to mechanically block antennal movements (Batchelor and Wilson, 2019; Budick et al., 2007; Camhi and Johnson, 1999; Chatterjee et al., 2022; Duistermars et al., 2009; Mamiya and Dickinson, 2015; Natesan et al., 2019; Robie et al., 2010; Sun et al., 2009; Suver et al., 2019, 2023). Despite this body of research, there remain gaps in our understanding of how antennal mechanosensation guides behavior; future studies aimed at filling in these gaps will benefit from a detailed method to block antennal sensation.

Here, we synthesized a reliable and reproducible method for mechanically blocking antennal movements in *D. melanogaster*. When glue is applied to the passive joint (A2/A3), A3 is unable to rotate about its long axis, thereby preventing JON activation. Although there are various tools available for genetically silencing specific subsets of neurons such as the JONs, these methods often require additional genetic crosses to incorporate them into an experimental fly line and can suffer from complications such as off-target expression. In contrast, mechanically blocking the antennae can be immediately applied to adult flies of any genetic background. Further, unlike some optogenetic silencing techniques, which can introduce additional confounding variables such as heat and/or light effects, gluing the antennae avoids these issues. Altogether, this protocol prevents antennal movements, significantly reducing the detection of mechanosensory stimuli such as sound and wind.

## MATERIALS AND METHODS

### Fly husbandry

The flies (*Drosophila melanogaster* Meigen 1830) used in this study were raised on standard cornmeal molasses food and were maintained in an incubator at 25°C on a 12 h light/dark cycle. Adult female flies between 3 and 10 days old were used for the tethered behavior and multiphoton experiments here, but both male and female flies can be used with this protocol. For all tethered behavior experiments, we used a common genetic silencing control genotype (*Canton-S x UAS-GtACR1*; Bloomington no. 92983). The experiments presented here do not involve any optogenetic manipulation and instead focus on antennal kinematics, which are not expected to be influenced by the presence of the optogenetic effector (*UAS-GtACR1*), as it remains unexpressed without its corresponding transcription factor (e.g. GAL4). For all multiphoton experiments, we used a genetic line expressing both a fluorophore and a calcium indicator in all JONs (*nan-GAL4*>*UAS-GcAMP7f,tdTomato*; Bloomington no. 24903 and GcAMP7f line courtesy of K. Nagel Lab, NYU School of Medicine, USA).

### Anesthesia station

**Fig. 2.**
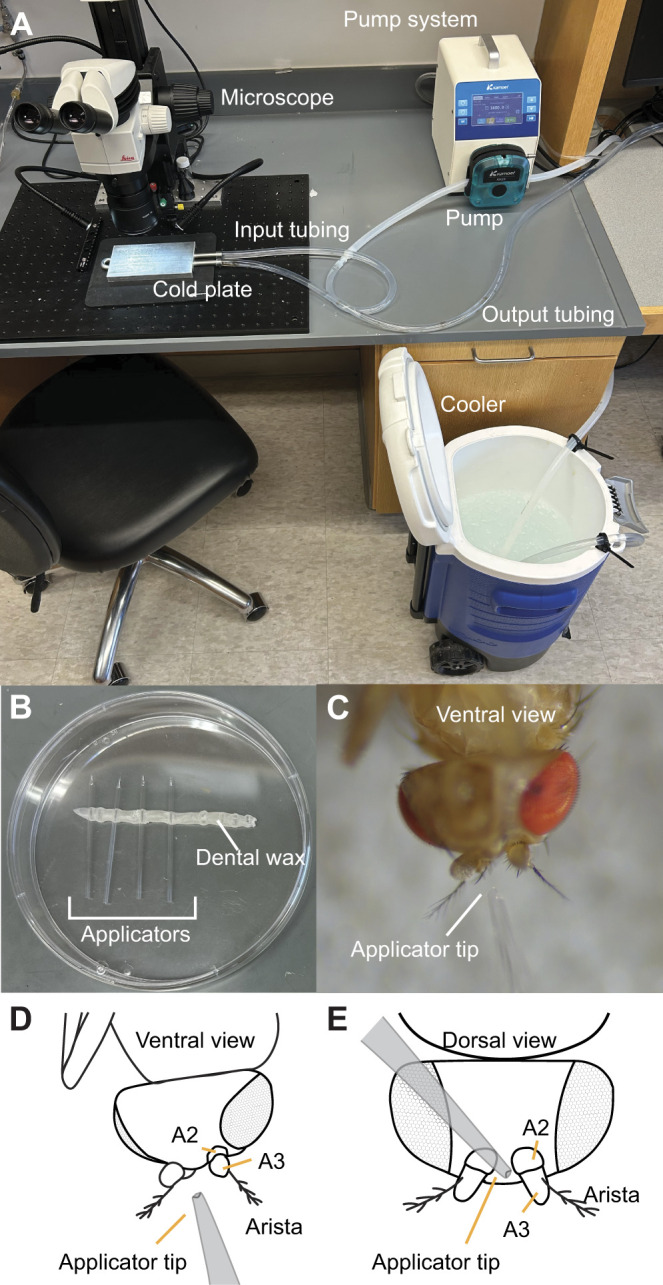
**Anesthesia station set-up and glue applicator reference.** (A) The anesthesia station set-up is used to keep flies cold-anesthetized, using ice water circulated through a stainless steel cold plate. (B) An assembled glue applicator storage container (Petri dish) with four glue applicators secured with dental wax. (C) A glue applicator tip next to the antennae. The tip is approximately a quarter of the diameter of A2. (D) A schematic diagram of the glue applicator tip next to the antennae from the ventral view (same view as shown in the photo in C). (E) A schematic diagram of the glue applicator tip next to the antennae viewed dorsally.

The anesthesia station (Fig. 2A) features a small peristaltic pump (ODM, Kamoer) to -KK25 continuously circulate ice-cold water from a 5 gallon (∼19 l) cooler (00042115, Igloo, Katy, TX, USA) through pipes in a cold plate (35035K103, McMaster-Carr, Elmhurst, IL, USA). To make ice-cold water, we filled the cooler with pebble ice and added 2 gallons (∼7.5 l) of tap water. The cold water can be used immediately.

We submerged one end of a piece of tubing [i.d. 3/8 inch (∼9.5 mm), o.d. 9/16 inch (∼14.3 mm); 5233K64, McMaster-Carr) in the water reservoir held in the bottom of the cooler. After assembling the peristaltic pump to its pump system (AIPWIFI, Kamoer), we ran the other end of the tubing through the pump system and attached it to the input tube on the cold plate. We attached a second piece of tubing to the output tube on the cold plate and placed its other end in the cooler, completing the loop to recycle the ice water back into the cooler. The ice water will stay cold for at least 8 h if the cooler lid remains partially closed, resting lightly on the input and output tubing.

We placed the cold plate on a mouse pad (956-000035, Logitech) to provide insulation between the microscope base and the cold plate. We set the mouse pad and cold plate under a stereomicroscope capable of ×60 magnification (M80, Leica; equipped with a ×1.0 objective and ×10 eyepiece magnification; 10450910, Leica), so we could visualize key structures on the antennae (for example A2, which as measured along the dorsal–ventral axis, is about 0.12 mm in length). However, these structures are also visible under a stereomicroscope with ×50 magnification (SMZ745, Nikon; equipped with ×10 eyepiece magnification).

Although this protocol utilizes a small pump system to keep the flies at temperatures cool enough to anesthetize them, we recognize that a peristaltic pump can be expensive. A more affordable alternative could be implemented using an inexpensive Peltier system (e.g. as described in Loesche and Reiser, 2021), a fish tank pump, or by cooling the flies on a Petri dish placed on ice.

### Preparing the glue applicators

We attached one row of dental wax (40201616938, Fresh Knight) to the inside of a Petri dish (FB0875712, Fisher Scientific) to store the electrodes we used for gluing the antennae. Before we stuck the wax to the Petri dish, we doubled its length by gently rolling it between our hands lengthwise. We returned the lid to the dish after assembly to limit the amount of dust and debris that could enter the glue-applicator storage container.

We used thin-walled glass capillaries (TW150F-3, World Precision Instruments) and a micropipette puller (P-1000, Sutter Instruments) to create a generic long-tapered electrode program (e.g. as described in Oesterle, 2018). A recent 2-line electrode program that we used for pulling glue applicators had a RAMP value of 504 (Line 1 – heat: 503, pull: 0, velocity: 52, time: 250/Line 2 – heat: 503, pull: 0, velocity: 0, time: 250). Once the program was established, we used it to pull several sets of glue applicators. We transferred each set of pulled electrodes side by side, gently pressing them into the wax in the applicator storage container (Fig. 2B).

We used the glue applicators with a pipetting system featuring a p-200 pipette tip (23-150R, Genessee Scientific) inserted into one end of a 30 inch (762 mm) section of soft-walled tubing [i.d. 1/16 inch (1.6 mm), o.d. 1/8 inch (3.2 mm); 50-236-5866, Fischer Scientific] – the other end was reserved for the glue applicator later in the protocol. Roughly 30 inch (762 mm) sections of soft-walled tubing work well. Although we manually applied pressure to the pipette tip for gluing the antennae in this experiment because of the precision it grants (similar to applying pressure in whole-cell patch-clamping methods), it is also possible to execute this protocol without this step (described below).

### Anesthetizing the flies

We used a flow rate of 1600 ml min^−1^ and a speed of 142.9 rpm to circulate ice-cold water through the anesthesia station. We transferred our experimental flies from their food vial to a small scintillation vial and placed the scintillation vial on ice for 30 s, or until the flies stopped moving. Before we transferred the flies from the scintillation vial to the cold plate, we put a Kimwipe (06-666, Fisher Scientific) on the cold plate to absorb any accumulated condensation. If there was already condensation on the cold plate, bubbles would appear between the surface of the cold plate and the Kimwipe. We removed the bubbles by gently pushing them out of the edges of the Kimwipe with our fingertips. If the cold plate was dry, we added a small amount of deionized water to the Kimwipe (e.g. with a wash bottle; Z423335-1PAK, Sigma-Aldrich) to adhere it to the cold plate and ensure it became cold. We carefully transferred an anesthetized fly from the scintillation vial to the cold plate, gently positioning it ventral side up with a paintbrush (757063405371, Princeton) and dull forceps (we reused a refurbished well-used or damaged pair; 11293-00, Fine Science Tools). We then focused the field of view onto the joint of interest; for example, the joint between the pedicel (A2) and the funiculus (A3).

### Loading glue into the applicator

After inserting the flat (non-sharp) end of the glass applicator into the open end of the applicator tubing (sharp end facing out), we gently broke the tip of the applicator by pushing the tip through a Kimwipe folded in half. Pushing the applicator through the Kimwipe twice usually created a desirable size for glue application to the antennae, but sometimes it required a third push. Checking the size of the tip under a microscope, and in relation to the size of the fly's antennae (best observed when the fly is positioned ventral side up), helped to gauge an appropriately sized applicator tip, which was around a quarter of the diameter of the second antennal segment, or about 0.03 mm (Fig. 2C–E).

We then transferred a small drop of UV resin hard glue (1121S3, Wayin) to a microscope slide (12550003, Fisher Scientific) stored in a light-proof container (e.g. a Petri dish wrapped in aluminium foil; 458742928317, Reynolds). To load glue into the applicator, we applied suction to the applicator pipette tip after placing the sharp tip of the applicator into the drop of UV glue, taking care to not insert the glass tip so far that we crushed it on the surface of the microscope slide. We stopped suction before pulling the applicator out of the glue to limit pulling air bubbles into the applicator, and we only loaded enough glue to fill the tip of the applicator.

As an alternative to applying the UV glue with pressure, the glue is viscous enough to form a small droplet on the exterior surface of the applicator tip and can be directly transferred to the fly's antennae. However, we found that this method increases variability in the amount of glue applied to the antennae, often resulting in larger amounts than desired for minimal gluing of a single antenna joint.

### Gluing of the antennae

**Fig. 3.**
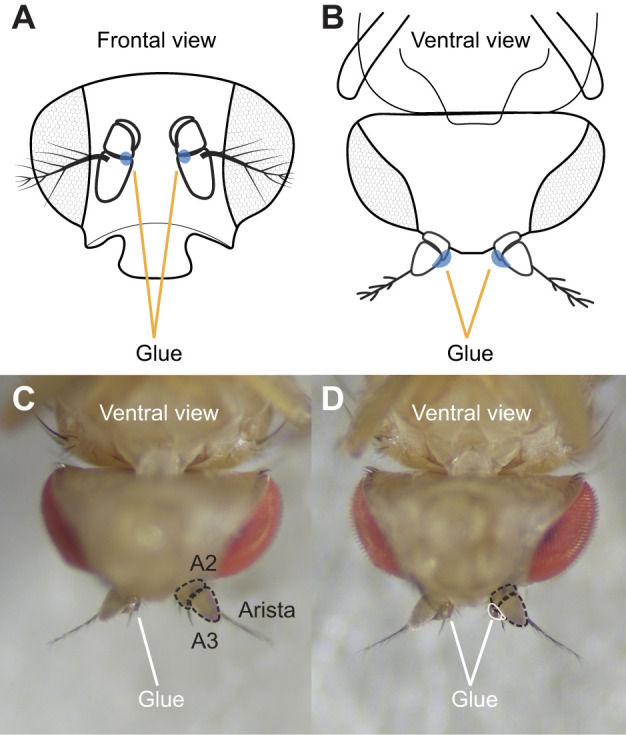
**Examples of glued antennae.** (A) Two schematized glue drops (blue circles) on the antennae viewed frontally. Glue is placed on the inside (medial) edge of the antenna. (B) Schematic diagram of glued antennae as viewed ventrally. (C) Example where only the antenna on the left is glued at the A2/A3 joint. Dashed black lines outline A2 and A3 segments on the right antenna. (D) Example where the A2/A3 joint on both antennae are glued. The white outline on the right antenna indicates the size and position of glue applied to the A2/A3 joint.

Using the glue applicator, we placed a small drop of UV glue on the joint (e.g. between A2 and A3, on the inside/medial edge of the antenna; Fig. 3A). However, during application, the glue frequently adhered to both the antenna and the applicator, leading to unfavorable positions for curing. To prevent this, we found it helpful to use a small paintbrush to gently hold the fly in a static position during glue application, ensuring better control and accuracy. When gluing the A2–A3 joint, placing the drop of glue on the side opposite the arista on A3 (i.e. the most medial part of this joint; Fig. 3B,C) minimized the risk of damaging the arista. Because some glue remained on the outside of the applicator after loading, care was taken not to touch any part of the fly other than the desired antennal joint. If glue contacted any unintended area, the fly was discarded and the procedure repeated with a new one.

After applying UV glue to the A2/A3 joint, we shone a UV flashlight (726972490877, MOLAER) on the fly for 15–30 s to cure the UV glue. To ensure the A2/A3 joint was glued, we used a single bristle sticking out from the side of a paintbrush to gently push the antenna with cured UV glue (Movie 1). Even without successful joint adhesion, a gentle push with a single hair of a paintbrush or a gentle breeze produced by the experimenter wafting their hand near the fly readily induced motions of the A3 segment. To ensure activity of the JONs was blocked throughout experiments, we checked the glued segments before and after experimentation.

**Fig. 4.**
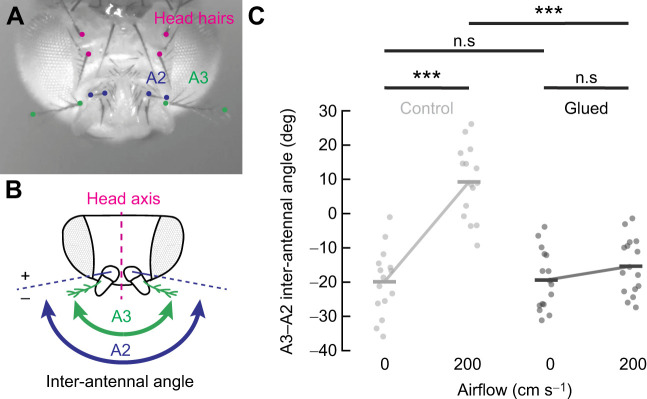
**Measuring passive antennal movements in response to airflow with a blocked A2/A3 joint.** (A) Example video frame with tracked parts on the antennae and head. (B) Labeled points are grouped to calculate inter-antennal angle. Movements of A3 (green) relative to A2 (blue), the passive joint, were measured. (C) Passive joint angle during airflow in flies with antennae free (control; light gray) and with A2/A3 glued (dark gray). Dots indicate the average inter-antennal angle across trials per fly (*n*=10 trials per fly; *N*=15 flies control, *N*=16 flies glued), and bars indicate group means across all flies. Airflow at 200 cm s^−1^ significantly increased A3–A2 angle compared with that at 0 cm s^−1^ in control flies (***P<0.0001; Tukey–Kramer), and this effect was abolished when the A2/A3 joint was stabilized with glue (*F*_1,1_=28.35, ***P<<0.0001; two-way ANOVA). No significant difference was observed between control and glued groups at 0 cm s^−1^.

**Fig. 5.**
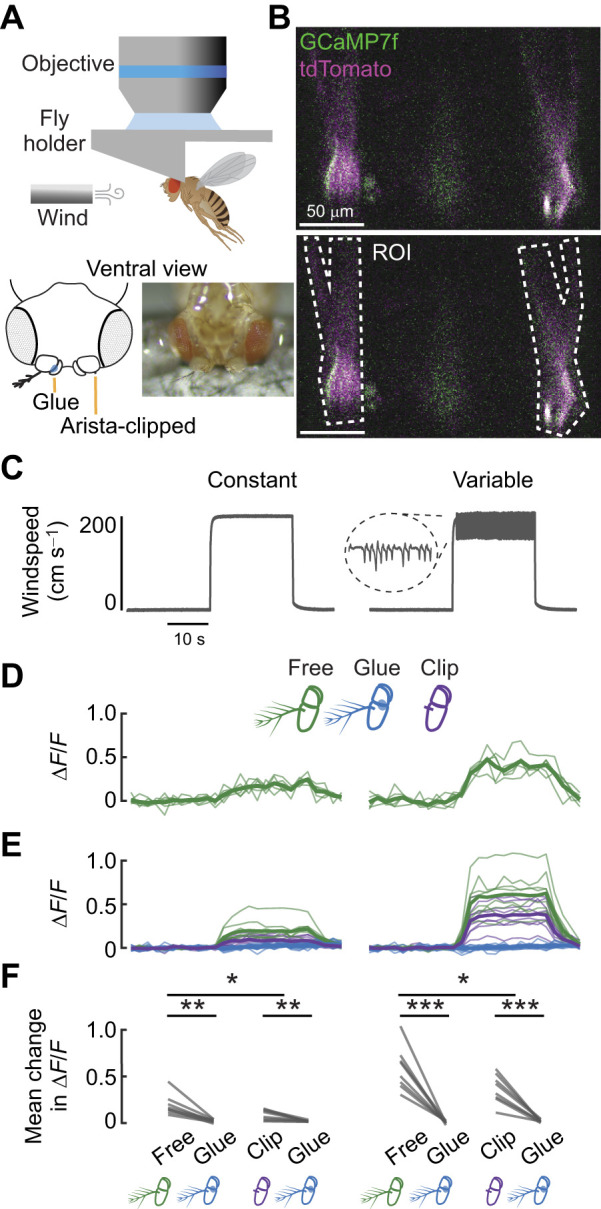
**JON responses to airflow following A2/A3 immobilization and arista removal.** (A) Top: schematic drawing of the two-photon imaging experimental setup with frontal airflow directed towards the fly. Bottom: schematic diagram and image of one experimental fly, as viewed ventrally, with a glued left antenna and a clipped right arista. (B) Average intensity projection image of JON axon terminals for both brain hemispheres. Regions of interest (ROI) are outlined in white. (C) Constant (left) and variable (right) speed pulses of airflow presented to the fly during imaging. A 300 ms magnified portion of the variable stimulus is shown inside the dotted ellipse. (D) Normalized JON response (normalized fluorescence, Δ*F*/*F*) to constant (left) and variable (right) airflow across *n*=5 trials in one fly (thin lines) where the antenna is free. The mean across trials is represented by the thicker line. (E) Normalized response to constant (left) and variable (right) airflow in JONs with free antenna (green), A2/A3 immobilized (blue) and arista-clipped antenna (purple) treatment groups. Thin lines represent the mean of all trials for each fly, and thicker lines represent the mean across flies (*N*=8 flies, *n*=40 trials for free and clipped treatment; *N*=16 flies, *n*=80 trials for glued treatment). (F) Mean change in normalized fluorescence in response to constant (left) and variable (right) airflow. Each line represents the paired response within an individual fly (paired two-sample *t*-test: for the constant stimulus, free–glue comparison was **P=0.002 and clip–glue comparison was **P=0.009; for the variable stimulus, free-glue comparison was ***P<0.001 and clip–glue comparison was ***P<0.001). In the clipped arista treatment, JON activity was also compared with that in the free antennae treatment (independent two sample *t*-test, comparisons for the constant and variable stimulus were *P=0.032 and *P=0.042, respectively).

Here, we applied this protocol to block A2/A3 movements and significantly reduce airflow sensitivity in tethered flies, which we validated using kinematic tracking of the antennae (Fig. 4) and two-photon functional imaging of JON terminals in the central brain (Fig. 5). Additionally, we provide data that suggest mechanically blocking the A2/A3 joint, as described in this protocol, is more effective at reducing JON activity than clipping the arista alone (Fig. 5).

### Two-photon functional calcium imaging

To test the effectiveness of the gluing procedure, we imaged activity in sensory neurons (JONs) in tethered flies using a Thorlabs 2-photon microscope equipped with galvo-galvo scanning mirrors, a 920 nm laser (Axon Coherent) and an apochromatic near-infrared water-immersion objective (Nikon, ×40 magnification, 0.8 NA, 3.5 WD). Each fly had one free antenna and the other glued, or one glued antenna and the other with its arista clipped. We alternated the antennal treatments between the fly's left and right antenna. We collected fluorescence signals using GCaMP7f (calcium indicator) and tdTomato (cell marker) in the JON axon terminals. The field of view we selected captured both hemispheres simultaneously with a ∼0.5 Hz scanning rate (∼1.867 s frame^−1^ capture rate), ×1.4 zoom, and a 288×192 pixel resolution (237.78×158.52 μm). For each fly, we captured a whole volume (60–100 μm) of the JONs in 0.5 μm steps, from which we selected an imaging plane approximately halfway along the *z*-axis.

We measured activity during a baseline period of 20 s without airflow, followed by 20 s of airflow, and another 10 s without airflow for each fly. We designed an airflow delivery system where a mass flow controller (6AGC1AL55-08AB, Dakota Instruments) controlled the speed of airflow through a stainless-steel tube (89895K657, McMaster-Carr) positioned approximately 1 cm anterior to the fly. To toggle airflow on and off, we used a solenoid valve (LHDA1233115H, Lee Company) controlled by a custom-written MATLAB script. During the stimulus period, we randomly alternated the airflow to be either constant (continuous at ∼190 cm s^−1^) or variable (between 120 and 205 cm s^−1^) to measure responses from JONs activated by a range of frequencies (i.e. low and high). We designed the variable stimulus to have 20 ms intervals where a random 80% of those intervals end with a solenoid valve closing briefly; this length of time was randomly determined between 0.4 ms and 1.5 ms. To measure airflow speed, we used a calibrated hot wire anemometer (Dantec MiniCTA with 55P11 probe) mounted at the position of the fly's head.

### Data analyses

#### Kinematic antennal analysis

All data were collected in MATLAB (version 2023b, The MathWorks Incorporated) and analyzed using custom-written Python scripts (Python 3, version 3.10.11, Python Software Foundation). We used DeepLabCut (version 2.3.6; Mathis et al., 2018), to track antennal movements over time, and a ResNet-50 neural network trained on 670 frames from 67 flies. The network was trained over 500,000 epochs with a training/test fraction of 0.8. *Post hoc* evaluation of the network revealed a training error of 1.94 pixels and a testing error of 2.24 pixels. We used Kolmogorov–Smirnov tests to test for normality in all behavioral groups.

#### Two-photon functional calcium imaging

All data were collected using ThorsImagesLS (version 4.3) and MATLAB (version 2024a, The MathWorks Incorporated) and analyzed using custom-written MATLAB scripts. We manually determined the regions of interest (ROI) in each fly by visually inspecting the maximum intensity projection of the tdTomato signal and outlining two ROI – one for each hemisphere of JONs. We subtracted the background for each fluorescence signal, defined as the lowest 20% of pixel intensity values in the entire field of view, from both signals. The GCaMP7f signal was normalized to the tdTomato signal to account for brain movement in the *z* dimension. We calculated time-dependent fluorescence signal (*F*_t_) by averaging the GCaMP7f signal within the ROI for each acquisition frame. Following this, we calculated the time-independent baseline fluorescence (*F*_0_) by averaging the GCaMP7f signal across the pre-stimulus window. We calculated the change in fluorescence (Δ*F*) as the difference between the fluorescence signal and the pre-stimulus baseline, relative to the baseline fluorescence, or Δ*F*=(*F*_t_−*F*_0_)/*F*_0_. To quantify the change between the pre-stimulus and during-stimulus activity, we subtracted the mean Δ*F* of the first 20 s (10 frames) before the onset of the wind stimulus from the mean Δ*F* during the last 15 s (7 frames) of the wind stimulus for each trial.

## RESULTS AND DISCUSSION

After the successful application of glue at a joint between two antennal segments (e.g. A2 to A3), these segments will move in unison. An experimenter can validate this by using a paintbrush with a single bristle sticking out to the side (or a brush trimmed to a single bristle) to gently move the glued antenna and visually observe the two joints moving together. We recommend checking the glued segments before and after experimentation, to ensure movement of the desired antennal joint is blocked throughout experiments.

### A2/A3 joint immobilization blocks passive antennal deflections caused by airflow

To quantitatively validate our gluing protocol's ability to mechanically block movements of A3 relative to A2, we compared antennal movements in flies with and without their A2/A3 joint glued. We presented airflow to non-flying pin-tethered flies with and without their A2/A3 joint glued.

We presented two windspeeds, 0 cm s^−1^ and 200 cm s^−1^, for 6 s of constant airflow, with averages taken over the last 4 s. Each fly was presented with 10 trials consisting of both windspeeds in a randomized presentation order. We recorded antennal movements with machine vision cameras (E0030003, Allied Vision) and analyzed antennal movements with DeepLabCut (Mathis et al., 2018) and custom-written Python scripts. We tracked 12 points on the head and antennae of the fly, including the base of four static head hairs to determine the midline, the tip and base of the arista to define the passive movements of A3, and the tip and base of a hair on A2 to define active movements (Fig. 4A).

We then measured the displacement of A2 and A3 relative to the medial head axis, and, for each trial, we summed the left and right antennal angles together to obtain the inter-antennal angle (i.e. the angle between the two antennae) for A2 and A3 (Fig. 4B). We analyzed these results across all trials for each fly and compared the average response of each fly across each experimental condition: with and without glue at 0 cm s^−1^ (no airflow) and 200 cm s^−1^ (Fig. 4C). Data are presented as the angle of A3 relative to A2.

We performed a two-way ANOVA to analyze the effect of airflow and gluing of the A2/A3 joint on the positioning of A3 relative to A2. The ANOVA revealed a statistically significant interaction between the effects of airflow and gluing the A2/A3 joint (*F*_1,1_=28.35, *P*≪0.0001). Additionally, we performed a *post hoc* Tukey–Kramer analysis, which indicated a statistically significant difference in relative A3 positioning across windspeeds in non-glued flies (*P*≪0.0001) and across gluing conditions in the presence of 200 cm s^−1^ wind (*P*≪0.0001). This suggests that gluing the A2/A3 joint significantly reduced passive antennal deflections in response to airflow.

### A2/A3 joint immobilization silences JON activity

To measure the effect of joint immobilization on mechanosensory activity, we performed functional imaging in flies with different antennal manipulations exposed to wind stimuli (Fig. 5A). We conducted within-animal comparisons for two pairs of antennal manipulations: glued versus free and glued versus arista-clipped antenna (Fig. 5A). We recorded activity in the JON axons, which terminate in the antennal mechanosensory and motor center in the central brain (Kamikouchi et al., 2006). We recorded calcium activity of the JONs, normalized the signal to that of a co-expressed fluorescent cell marker in the JONs (Fig. 5B), and calculated the change in calcium activity before and during the airflow stimulus.

In response to both constant and variable wind stimuli (Fig. 5C), JON activity in free antennae increased (Fig. 5D) as expected based on previous studies of JON activity (Patella and Wilson, 2018). In contrast, antennae with the A2/A3 joint glued did not exhibit a large increase of JON activity in response to either wind stimulus. After joint immobilization, JON activity was minimal during both constant and variable airflow stimuli but not entirely abolished (Fig. 5E, constant and variable airflow elicited increases in the Δ*F*/*F* signal by 1.7% and 0.9%, respectively; one sample *t*-test, *P*<0.001; *N*=16 flies, *n*=80 trials). Importantly, the gluing method substantially reduced the change in JON activity in response to airflow stimuli compared with the free antenna (Fig. 5F; paired two sample *t*-test, *N*=8 flies, *n*=40 trials; constant stimulus, *P*=0.002; variable stimulus, *P*<0.001). Compared with that of the free antennae, the gluing method reduced the JON activity in response to the constant stimulus by 90±3.1% on average, and the variable stimulus by 99±1.1% on average, about 95% overall. Together, these data suggest that gluing the A2/A3 joint successfully minimizes JON sensitivity to airflow stimuli.

An alternative strategy to reduce antennal mechanosensation includes clipping the arista, so we compared this method with our joint gluing method (Fig. 5). Under the clipped arista treatment, JON activity was significantly lower than that of the free antennae treatment across flies (Fig. 5E,F; independent two-sample *t*-test, *N*=16 flies, *n*=40 trials; constant stimulus *P*=0.0320; variable stimulus *P*=0.0424). Compared with that of the free antennae, the clipping method reduced the average JON activity in response to the constant stimulus by 55%, and the variable stimulus by 38%. Although clipping the arista reduced activity in the JONs, the average decrease in JON activity with either airflow stimulus was significantly lower in the glued joint treatment than in the clipped arista treatment within flies (Fig. 5E; paired two-sample *t*-test, *N*=8 flies, *n*=40 trials; constant stimulus *P*=0.009, variable stimulus *P*<0.001). Compared with clipping the arista, the gluing method reduced the JON activity in response to the constant stimulus by 70±7.1% on average, and the variable stimulus by 95±1.7% on average (Fig. 5F). Taken together, we found that arista clipping produces a moderate reduction in JON sensation, and that A2/A3 immobilization significantly reduces JON sensory activity. Thus, we conclude that the gluing method described here is an effective method to block antennal mechanosensation.

### Limitations and potential solutions

This protocol precisely immobilizes specific antennal joints; however, there are some limitations of this method. After gluing, the exact angle of A3 relative to A2 may vary slightly from fly to fly in these experiments, as it is difficult to precisely control the final angle during the gluing procedure. Subtle variations in A3 position could tonically stretch and activate subpopulations of JONs, so the experimenter should attempt to glue the joint as close to its resting posture as possible to minimize this. Additionally, this protocol does not directly control for the volume of glue added to each antenna. Larger quantities of glue could potentially weigh down the antennae and effect active antennal movements. Thus, the experimenter should use as little glue as necessary to secure the A2/A3 joint and should attempt to apply the same amount of glue to both antennae. Additionally, ambient light from the environment can begin to cure the glue before application, increasing the viscosity of the glue over time; to avoid this, the experimenter can store the glue slide in a light-proof container (such as a small Petri dish covered in aluminium foil) when performing the gluing procedure. Using UV glue with curing sensitivity at a lower wavelength or conducting this protocol in a dim or windowless room could also help mitigate this.

Flies also frequently groom their antennae with their legs and will groom furiously to attempt to dislodge a drop of glue at the A2/A3 joint (if the legs are left intact), as described in a previous study (Suver et al., 2019). If the glue does not adhere to the joint for the duration of the experiment, using a less viscous glue will help ensure the glue seeps further into the joint and prevent dislodging during grooming.

### Conclusion

In this study, we detailed a protocol for gluing antennal joints in *Drosophila*, thereby mechanically blocking activity of JONs. Although this protocol describes blocking the A2/A3 joint in detail, it is precise enough to be applied to other joints; for instance, the joint between the scape (A1) and pedicel (A2). Our protocol provides precise details for a classic antennal stabilization technique, enabling reliable reproducibility with step-by-step instructions, limitations to consider and potential solutions. We also provide helpful references that use gluing methods to block JON activity in flies and in other insects (e.g. hawkmoths and cockroaches; Batchelor and Wilson, 2019; Budick et al., 2007; Camhi and Johnson, 1999; Chatterjee et al., 2022; Duistermars et al., 2009; Mamiya and Dickinson, 2015; Natesan et al., 2019; Robie et al., 2010; Sun et al., 2009; Suver et al., 2019, 2023).

## Supplementary Material

10.1242/jexbio.251495_sup1 Supplementary information
