# Supplementary figures and images for: A method for mechanically blocking antennal joints in Drosophila
Source: J Exp Biol. 2025 Nov 21;228(22):jeb251495. doi: 10.1242/jeb.251495 (PMC12669834; doi:10.1242/jeb.251495)

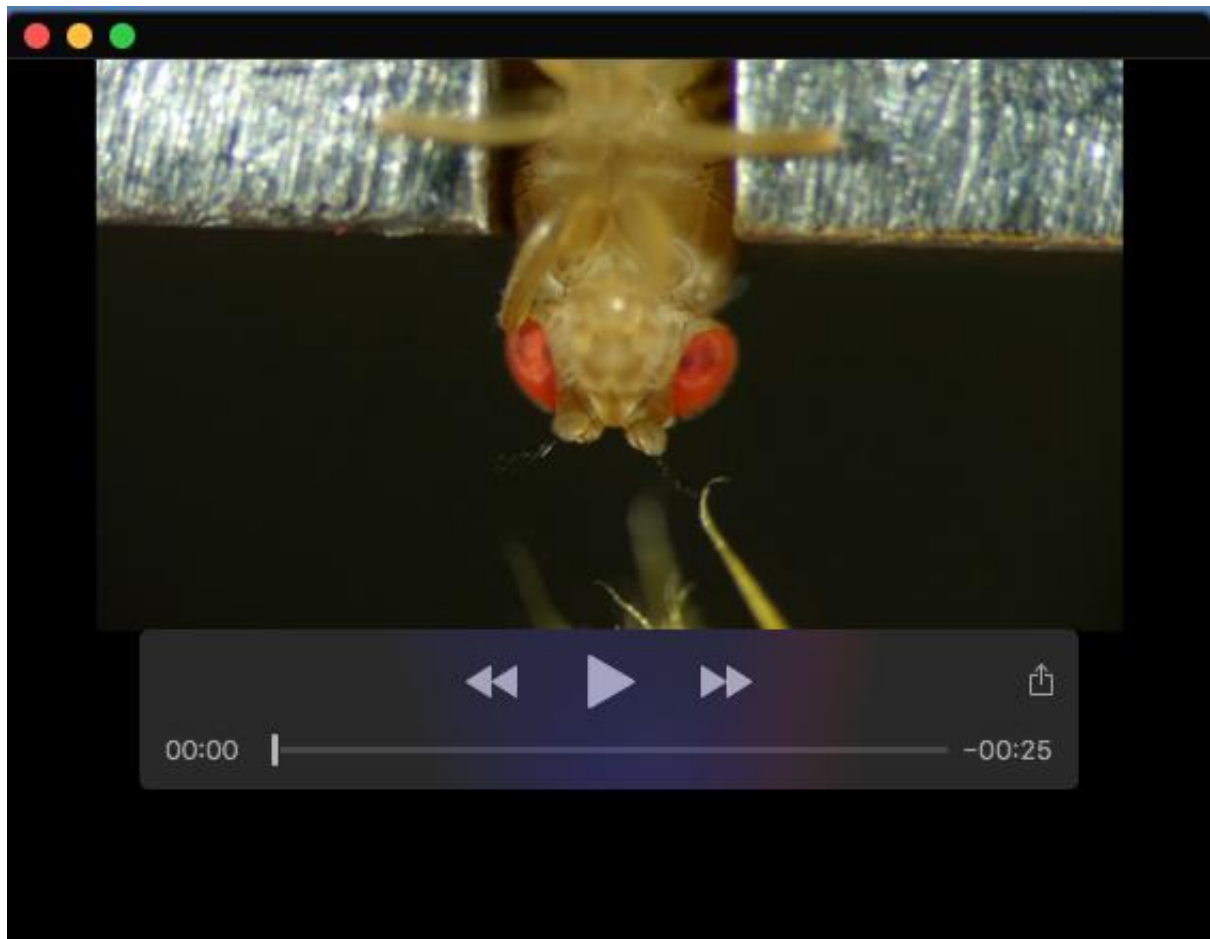

**Movie 1.** Comparison of antennal movements in free antenna versus antenna with A2/A3 glued joint.

Supplement: Supplementary information [file jexbio-228-251495-s1.pdf]
